# Behavior and Three-Dimensional Finite Element Modeling of Circular Concrete Columns Partially Wrapped with FRP Strips

**DOI:** 10.3390/polym10030253

**Published:** 2018-03-01

**Authors:** Junjie Zeng, Yongchang Guo, Lijuan Li, Weipeng Chen

**Affiliations:** School of Civil and Transportation Engineering, Guangdong University of Technology, Guangzhou 510006, China; jjzeng@gdut.edu.cn (J.Z.); lilj@gdut.edu.cn (L.L.); 18819457038@163.com (W.C.)

**Keywords:** finite element (FE) analysis, plastic-damage model, circular column, FRP, partially FRP-confined concrete, confinement

## Abstract

Fiber-reinforced polymer (FRP) jacketing/wrapping has become an attractive strengthening technique for concrete columns. Wrapping an existing concrete column with continuous FRP jackets with the fiber in the jacket being oriented in the hoop direction is referred to as FRP full wrapping strengthening technique. In practice, however, strengthening concrete columns with vertically discontinuous FRP strips is also favored and this technique is referred to as FRP partial wrapping strengthening technique. Existing research has demonstrated that FRP partial wrapping strengthening technique is a promising and economical alternative to the FRP full wrapping strengthening technique. Although extensive experimental investigations have hitherto been conducted on partially FRP-confined concrete columns, the confinement mechanics of confined concrete in partially FRP-confined circular columns remains unclear. In this paper, an experimental program consisting of fifteen column specimens was conducted and the test results were presented. A reliable three-dimensional (3D) finite element (FE) approach for modeling of partially FRP-confined circular columns was established. In the proposed FE approach, an accurate plastic-damage model for concrete under multiaxial compression is employed. The accuracy of the proposed FE approach was verified by comparisons between the numerical results and the test results. Numerical results from the verified FE approach were then presented to gain an improved understanding of the behavior of confined concrete in partially FRP-confined concrete columns.

## 1. Introduction

**Figure 1:**
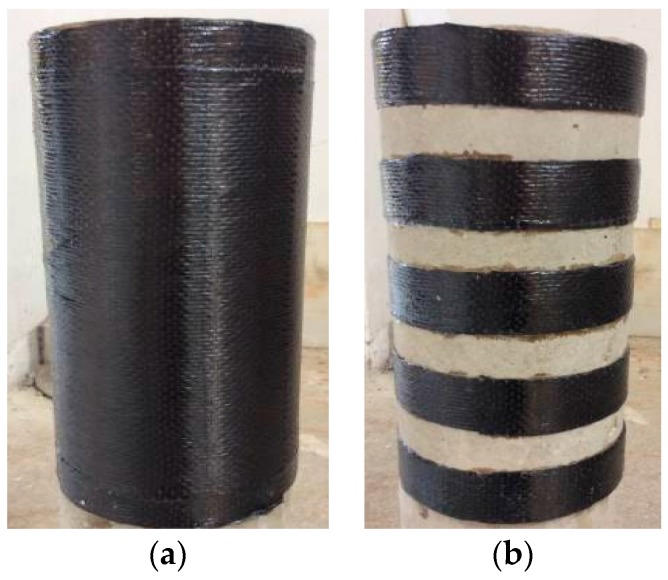
Fully and partially FRP-confined concrete. (**a**) Fully FRP-confined concrete; (**b**) Partially FRP-confined concrete.

Over the past two decades, fiber-reinforced polymer (FRP) has become a favorite material for the strengthening of existing concrete columns. The FRP strengthening technique for a concrete column is predominantly referred to as FRP full wrapping strengthening technique in which an existing deteriorated concrete column is fully wrapped with FRP jacket along the height of the column (Figure 1a). The resulting column of this strengthening technique is referred to as a fully FRP-confined concrete column. Existing research has demonstrated that the compressive strength and ductility of circular columns fully wrapped with FRP jackets are substantially enhanced [1,2,3,4,5,6,7,8,9,10,11,12,13,14].

Alternatively, partially FRP-confined concrete columns, usually wrapped with FRP strips, refer to FRP-confined concrete columns with discontinuous FRP jackets along their longitudinal axis (Figure 1b). Strengthening existing columns by FRP partial wrapping are hereafter referred to as FRP partial wrapping strengthening technique. Although most studies related to FRP-confined concrete columns are on fully FRP-confined concrete columns, partially FRP-confined concrete columns have also been demonstrated to possess adequate increase in strength and excellent increase in ductility compared with its corresponding un-confined columns (e.g., [15]). Under certain circumstances, partially FRP-confined concrete columns are comparatively favorable, especially for columns only requiring considerable increase in axial deformation capacity. Additionally, less FRP materials are required for partially FRP-confined concrete columns while FRP partial wrapping strengthening technique can be applied more easily and faster than FRP full wrapping strengthening technique [16]. The interest in understanding the behavior of partially FRP-confined concrete has led to several experimental studies on partially FRP-confined concrete columns [15,17,18,19,20,21,22,23,24,25]. The research has demonstrated that the FRP partial wrapping increases both strength and ductility of concrete. The existing test results enable the verification and assessment of available design guidelines for partially FRP-confined concrete columns [26,27] in which a longitudinal confinement effectiveness factor based on “arching action” [28,29] is suggested for partially FRP-confined concrete so that the partially FRP-confined concrete columns can be designed with appropriate accuracy.

**Figure 2:**
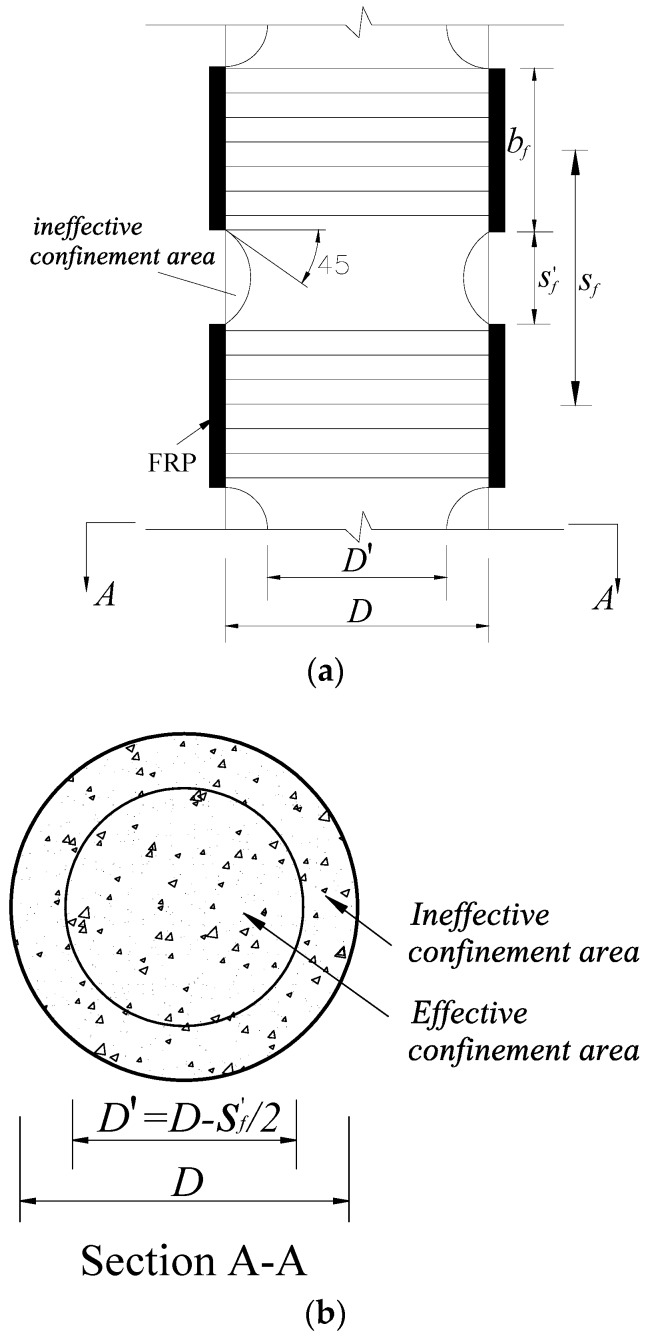
Effective confinement area in a circular column partially wrapped with FRP. (**a**) Arching action; (**b**) Effective confinement area at the center level of a strip.

**Figure 3:**
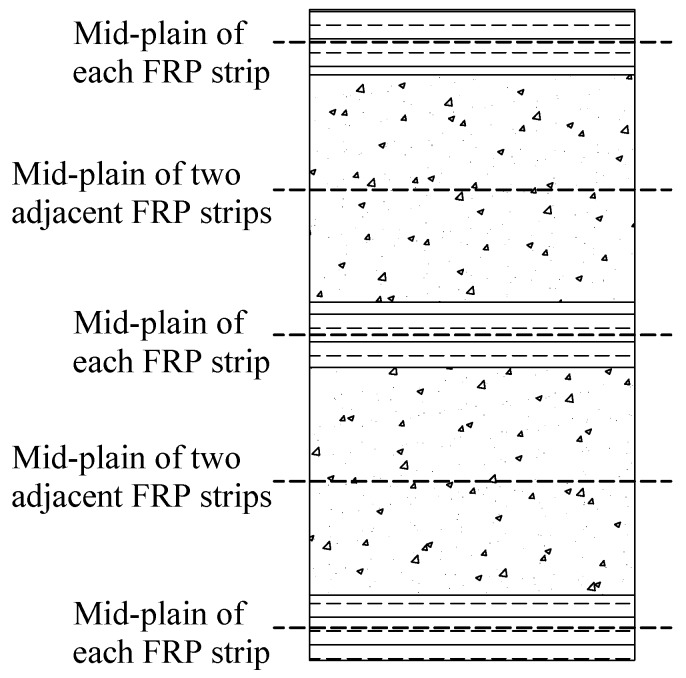
Illustration of the mid-plain of each FRP strip and the mid-plain of two adjacent FRP strips.

In real application, however, the lateral confinement can be provided by many different approaches, including simple transverse steel reinforcement (e.g., [28,29,30]), steel cage (e.g., [31,32]), steel tube (e.g., [33]), FRP (e.g., [20,34,35]), shape memory alloy (e.g., [36,37]), and active confinement techniques such as post-tensioned metal straps (e.g., [38,39]). In these structures, concrete receives either uniform or non-uniform confinement from its confining mechanism. In particular, the confinement to the confined-concrete in fully wrapped axial loaded circular columns is uniform. However, concrete under non-uniform confinement is more commonly seen in practice, such as FRP-confined concrete in square columns or confined concrete subjected to eccentric compression [40]. As for partially FRP-confined concrete in circular columns, the confinement is non-uniform along the height due to the usage of discontinuous FRP strips [29]. The consequence of the usage of discontinuous FRP strips is the less effective confinement between the two adjacent FRP strips, which was assumed to arise from the “arching action” between two adjacent confining strips/hoops (Figure 2). As a result, the axial deformation of partially FRP-confined concrete columns is believed to concentrate at or near the mid-plain of two adjacent FRP strips (Figure 3) where the confinement from the FRP strips is weak.

To obtain in-depth understanding of partially FRP-confined concrete, the strains and stresses along the generatrix (column height) need to be understood appropriately. The strains distribution along the column height can be tackled by several strain gauges or particle image velocimetry (PIV) technique. However, laboratory tests were incapable of clarifying the distribution of stresses (axial stresses and confining pressures) along the column height, because of the limitations of laboratory measurements. To overcome the obstacles associated with laboratory studies as noted previously, a robust three-dimensional (3D) finite element (FE) approach based on improved concrete plastic-damage model (CDPM) [41] was employed. Compared with some other 3D FE models (e.g., [42,43]), this 3D FE model was shown to be more reasonable and accurate in modeling of concrete under non-uniform confinement from an FRP jacket owing to the improved CDPM constitutive model. The improvement of this CDPM model included three main aspects [41,44]: a yield criterion related to the third deviatoric stress invariant, a confinement-dependent hardening rule and a confined-dependent non-associated flow rule. This model was successfully implemented in ABAQUS for the FE analysis of FRP-confined circular/square concrete columns and hybrid FRP-concrete-steel double-skin tubular columns under concentric compression [41]. The model has subsequently been utilized by some other researchers (e.g., [40,45,46,47]) and it was found that this model is capable of providing reasonable stress-strain responses for FRP-confined concrete under both uniform and non-uniform confinement [40,48].

Against the above background, an experimental program was conducted to study axial compressive behavior of circular columns wrapped with CFRP strips. In total, 15 columns were fabricated and tested. The PIV technique was utilized to measure strains in the specimens. The test results of the experimental program were presented and discussed. The improved CDPM was employed for FE analysis of partially FRP-confined concrete columns. The accuracy of the proposed FE approach was verified by comparing the numerical results and the test results. The axial and confining pressure distributions based on numerical results were presented to verify the existing “arching action” and to illustrate the confinement mechanics of partially FRP-confined concrete.

## 2. Experimental Program

### 2.1. Test Specimens

A total of 15 cylindrical column specimens were prepared and tested to investigate the behavior of partially FRP-confined circular columns. All specimens had a diameter of 150 mm and a height of 300 mm. The main column parameters investigated in present study are the FRP strip width, clear spacing of two adjacent FRP strips and FRP thickness. The column specimens were wrapped with different numbers of FRP strips (3, 4 and 5 strips) with different widths (i.e., 25, 30, and 35 mm) and thicknesses (1, 2 and 3 layers). The design of strip number and strip width led to nine different clear spacing ratios in present study (Table 1). Note that the ratio between the clear spacing of the two adjacent FRP strips and the center-to-center spacing of adjacent strips is referred to as clear spacing ratio ($s_{f}^{\prime }/s_{f}$) (Figure 1). The details about the test columns (including the clear spacing of the two adjacent FRP strips, FRP volumetric ratio and FRP thickness) are given in Table 1. Each column was given a name (Table 1), which starts with the letter “S” to represent “Specimen”, followed by a number indicating the number of FRP layer/layers. This is then followed by a number presenting the total number of FRP strips and then followed by the width of FRP strips.

### 2.2. Preparation of Specimens and Material Properties

Normal concrete made of river sand and natural gravels was utilized to cast the column specimens. Standard cylinders were also cast with the same batch concrete to determine the mechanical properties of the unconfined concrete. After the concrete columns had been cured at room temperature for 28 days, columns were wrapped with carbon FRP (CFRP) strips using a wet layup process, with fibers oriented only in the hoop direction. A 150-mm-long overlapping zone between the starting end and the finishing end of each strip could ensure the fully-developed tensile strength of the FRP.

Unidirectional high-tensile-strength carbon fiber sheets, with a nominal layer thickness of 0.167 mm, were utilized to form the FRP strips. FRP tensile tests were conducted to determine the material properties of FRP strips. In the tensile test, a unidirectional one-layer CFRP coupon was employed. The test procedure followed what is specified by the ASTM standard [49]. Five flat coupons were tested, and results were only based on coupons failed by FRP rupture at the middle. The average modulus of elasticity, tensile strength, and rupture strain of single-layer CFRP were found to be 249.1 GPa, 4477.6 MPa and 1.66%, respectively.

The concrete mix proportioning is 1:0.71:2.02:3.59 (cement:water:fine aggregate:coarse aggregate). The properties of unconfined concrete were obtained from compression tests on three standard concrete cylinders in accordance with ASTM C469 [50] and the unconfined concrete strength $f_{\mathrm{co}}^{\prime }$ and strain $\varepsilon_{\mathrm{co}}$ is determined by the compressive strength and strain of concrete cylinders (i.e., $f_{c}^{\prime }$ and $\varepsilon_{c}$). The compressive strength, strain at compressive strength and Poisson’s ratio of concrete cylinders were found to be 23.5 MPa, 0.0025 and 0.2, respectively.

### 2.3. Optical Strain Measurement

Particle image velocimetry (PIV) system is a novel digital correlation technique (hereafter this text will be abbreviated to PIV technique) that was originally developed in the field of experimental fluid mechanics by tracking the location of seed particles [51]. Based on the image-processing technique of normalized cross-correlation (i.e., principles of PIV), White et al. [52] implemented PIV in geotechnical tests (*geo*PIV) by tracking the texture (i.e., the spatial variation of brightness) of *geo*PIV test patches instead of seed particles. A displacement field can then be calculated from the correlation of a reference surface and a displaced or deformed surface. It is worth noting that validation experiments using the PIV code reported by White et al. [53] have demonstrated that the precision of this measurement technique is typically better than 1/10th of one pixel and the strain precision of the technique is a function of the size of the image, the resolution of the cameras used, and the chosen gauge length. Recently, some researchers [54,55,56,57] employed PIV technique to catch axial and hoop strain distribution in FRP jacket of FRP-confined concrete columns because this technique is capable of measuring strains in a patch with a certain area simultaneously.

**Figure 4:**
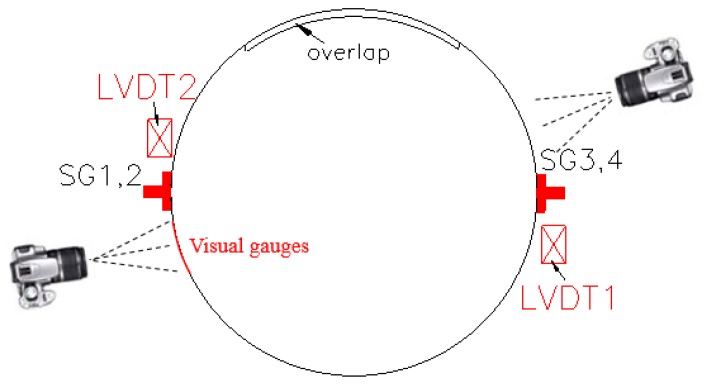
Location of cameras, foil strain gauges and LVDTs in the verification test.

**Figure 5:**
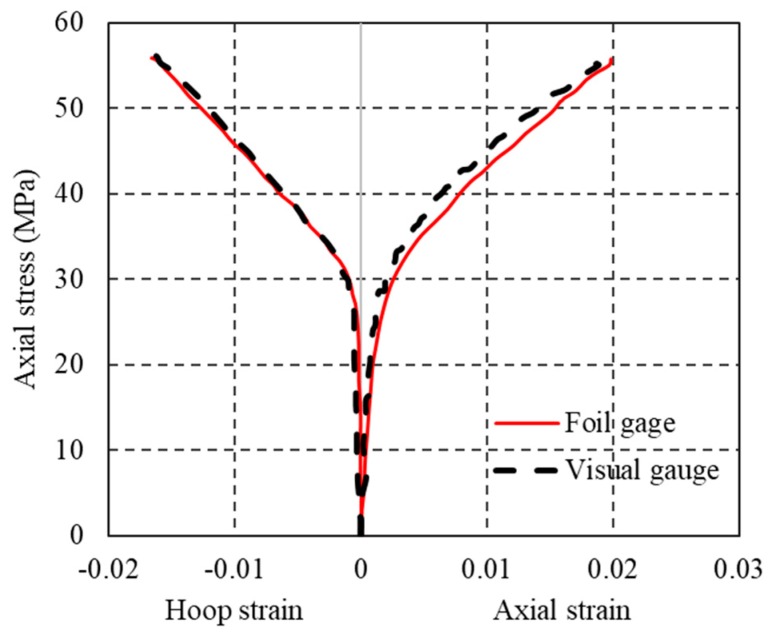
Stress-strain curves of FRP-confined concrete from visual and foil gauges.

To further validate the optical strain measurement technique for the current PIV application, a standard concrete cylinder (150 mm in diameter and 300 mm in height) fully confined with a one-layer CFRP jacket was tested under axial compression. In the test, both PIV technique and conventional strain measurement technique were utilized to measure axial and hoop strains: four strain gauges were installed on the FRP jacket at the mid-height: two at 180 degrees apart around the circumference were utilized to measure hoop strains and the other two were utilized to measure axial strains near the hoop strain gauges; two linear variable differential transformers (LVDTs) (TML, Tokyo, Japan) with a gauge length of 120-mm at 180 degrees apart were utilized to measure the axial shortenings (Figure 4). An overlap length of 150 mm was utilized in all these cylinders. An additional 25-mm-wide FRP strip was wrapped at each end of the cylinder to avoid premature local failure. The unconfined concrete strength and the axial strain at peak axial stress from three unconfined concrete cylinder tests were 28.9 MPa and 0.0025, respectively. The stress-strain curves of these CFRP-confined concrete cylinders from both the PIV and conventional sources are shown in Figure 5. Note that the axial strain was from the LVDTs and the visual strain gauges were located besides the conventional foil strain gauges (Figure 4). It is clearly seen from Figure 5 that the strains from both sources are in close agreement, demonstrating a good reliability of the PIV technique. The technique was thus employed in this study to measure the hoop and axial strain distribution of partially FRP-confined concrete columns.

**Figure 6:**
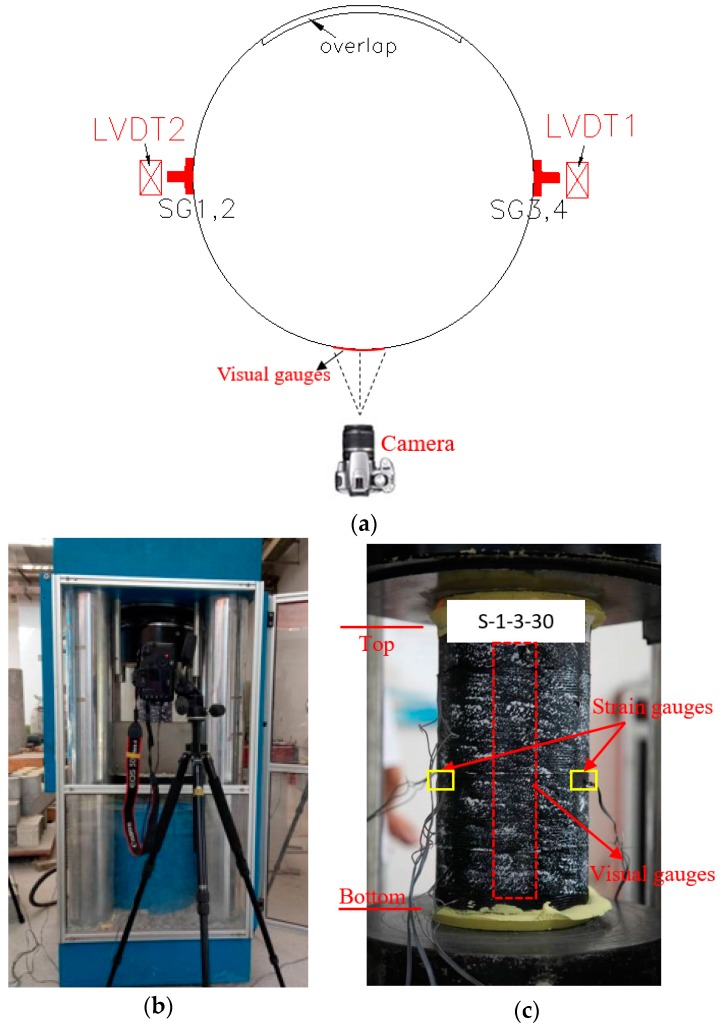
Instrumentations. (**a**) Location of cameras, foil strain gauges and LVDTs; (**b**) The system of PIV image acquisition; (**c**) Visual gauges and foil gauges.

Figure 6 provides schematic diagrams showing the instrumentations for the column tests. Digital images with the field of view shown in Figure 6a were captured every three seconds during the loading, employing a Canon 5D-Mark II digital camera (Canon, Tokyo, Japan). The camera was electronically synchronized by controlling the frame rate using a digital input/output channel which allowed the load/strain data acquisition system to trigger the cameras during testing. The horizontal line linking the center of the camera lens is perpendicular to the longitudinal physical axis of the specimen and the location of the camera is shown in Figure 6b. The system of PIV acquisition has been carefully adjusted before the formal test starts and any dislevelment and inclination were not allowed during the tests.

**Figure 7:**
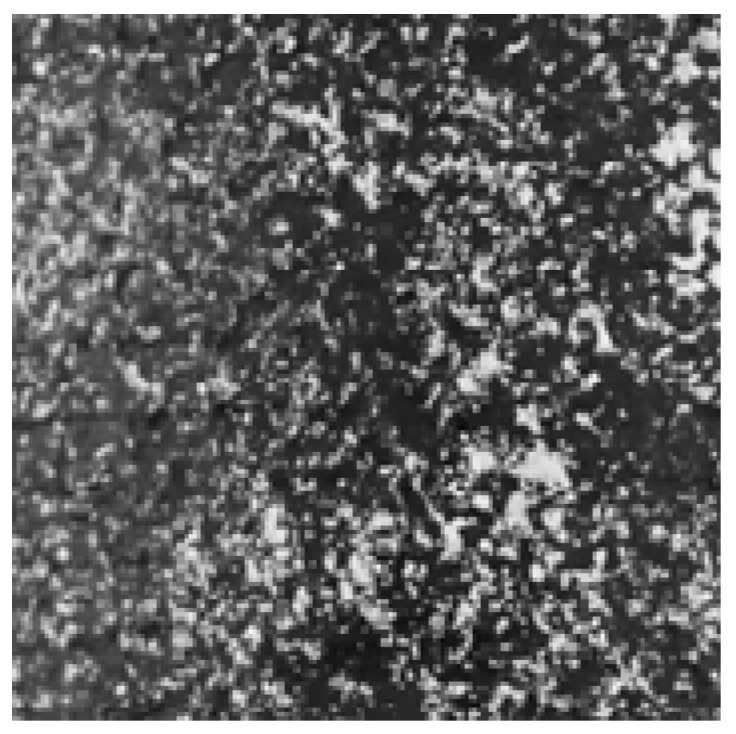
Particle texture on the specimen.

For the strain measurement using particle image velocimetry, an image-processing algorithm which uses normalized cross-correlation was utilized to calculate virtual axial and hoop strains along a vertical line for each of the three camera views. The details of the image analysis are not within scope of attention of current study, but essentially the technique defines regions of interest, called patches, in the first image of each set, and then used a specialized algorithm to track the displacements of each patch in subsequent images. A high-contrast image texture is required to ensure that each pixel patch contains sufficient pixel color variations for the algorithm to track successfully in subsequent images. The surfaces of these specimens were sprayed with black paint while the texture was formed by white paint. A schematic diagram showing details of the particle texture in the patch used for strain measurements is given in Figure 7.

### 2.4. Experimental Setup

All specimens were tested under concentric compression using a loading machine (SHIJIN, Jinan, China) with a load-carrying capacity of 5000 kN (Figure 6b). Each of the column ends were leveled using high-strength gypsum mortar so that the axial load could be simultaneously applied on the whole section. To ensure the centering of the specimen, each specimen was loaded to around 30% of its unconfined column strength to check the test alignment. Calibration was carried out by examining the readings from the two vertical strain gauges. The specimen was unloaded, realigned, and loaded again if the readings differ each other by 10% at the time the applied load reached about 30% of its unconfined column strength. Column compression test was realized using displacement control with a rate of 0.4 mm/min was used. In addition to the cameras, four strain gauges (HYCSYQ, Taizhou, China) were adopted to obtain the strains in FRP. Two of them were utilized to measure the hoop strains and the other two were employed to measure the axial strains, as is seen in Figure 6c. The full-height axial shortening was measured using the linear variable differential transformers (LVDTs) mounted in the testing machine. All test data, including the loads, displacements, and strains were recorded simultaneously by a data logger.

## 3. Test Results and Discussion

### 3.1. Failure Modes

**Figure 8:**
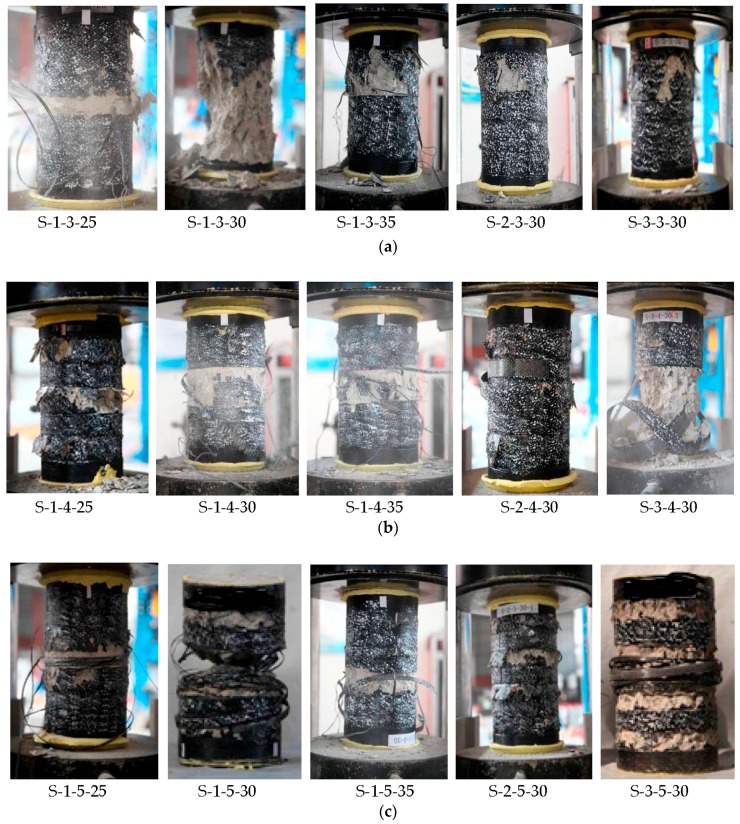
Failure modes. (**a**) Specimens wrapped with three FRP strips; (**b**) Specimens wrapped with four FRP strips; (**c**) Specimens wrapped with five FRP strips.

Figure 8 shows the failure models of the partially FRP-confined concrete specimens. As can be seen from the figure, all the specimens generally failed by the rupture of the FRP jackets. Some clicking sounds of epoxy rupture were first heard, followed by a sudden explosive sound of FRP rupture. FRP rupture occurred at or near mid-height and outside the overlap zone, with crushing of concrete simultaneously, resulting to a catastrophic failure with sudden loss of load capacity, which is in accordance with the experimental observations from Zeng et al. [15]. This failure did not take place at the column ends because of the existence of additional confinement from the additional strip at the column ends [48]. The failure initiated while concrete between adjacent FRP strips cracked, when the average axial stress approximately reached the unconfined concrete strength. Then the crack in the concrete grew and FRP rupture occurred, with crushing of concrete simultaneously. This failure point marks the ultimate condition of the FRP-confined concrete, as was widely adopted in previous study [14,58]. Likewise, the concrete crushing was more evident for specimens with larger clear spacing of adjacent FRP strips, implying concentration of localized failure of concrete between adjacent FRP strips where the confinement was low.

Specifically, the concrete cracking failure at mid-height of specimens wrapped with four FRP strips was more obvious than that of specimens wrapped with three and five FRP strips (Figure 8a,c). This is because the concrete at mid-height was not wrapped with any FRP strip for specimens wrapped with four FRP strips.

### 3.2. Stress-Strain Responses and Ultimate Condition

**Figure 9:**
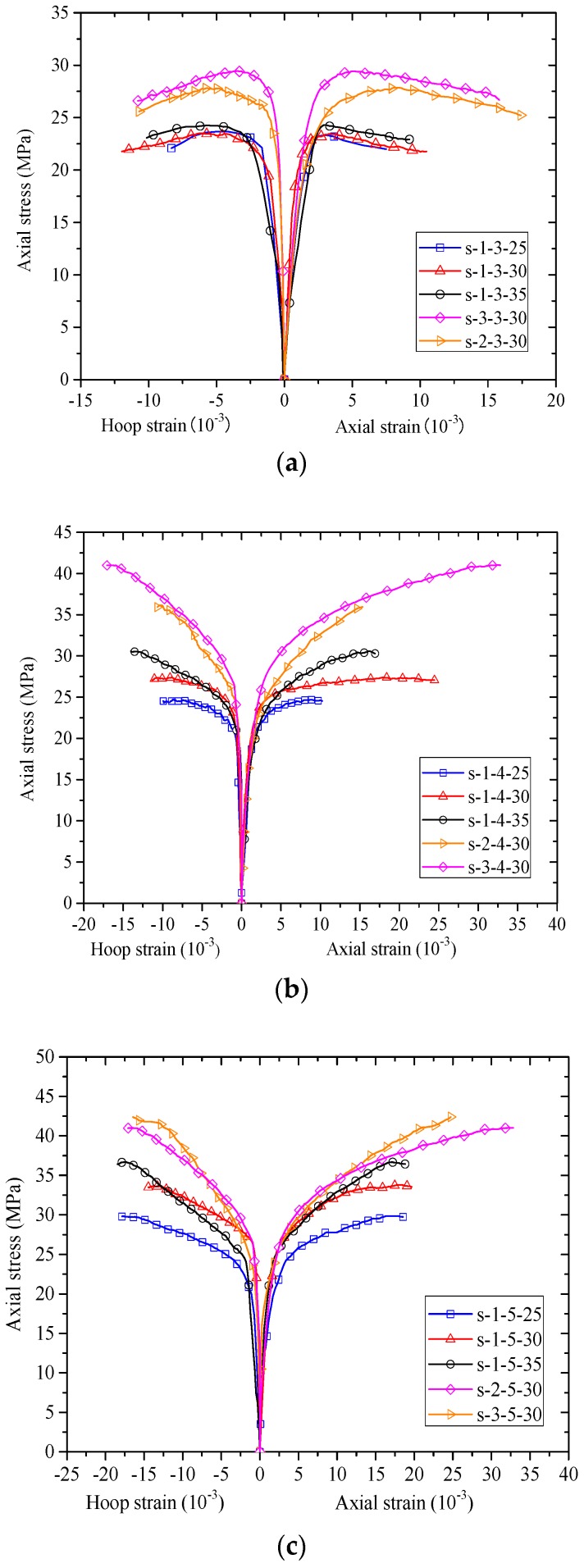
Stress-strain curves. (**a**) Specimens wrapped with three strips; (**b**) Specimens wrapped with four strips; (**c**) Specimens wrapped with five strips.

The stress-strain responses of the concrete in the test columns are shown in Figure 9. For stress-strain curves in present paper, the following sign convention is adopted: in concrete, compressive stresses and strains are taken to be positive, while in FRP jackets, tensile stresses and strains are taken to be negative. The axial stresses of concrete were calculated by dividing the axial load that it takes. The strain based on PIV technique is not discussed in the current section but it will be presented in the section below. In Figure 9, the axial strains were obtained from readings of the two LVDTs at the mid-height section. The axial strains from strain gauges were found to be much smaller than those from LVDTs due to the local failure of FRP jackets and thus they were not utilized [59,60]. The hoop strains were averaged from the two hoop strain gauges outside the FRP overlap zone at the mid-height section. In this article, the experimental stress-strain curves are terminated at the point when FRP rupture occurred, unless otherwise specified.

Figure 9 shows the stress-strain curves (i.e., axial stress-axial strain and axial stress-hoop strain curves) of concrete of the specimens. In each sub-figure of Figure 9, the number of FRP strips is identical, allowing direct comparisons between specimens with different FRP strip thicknesses and widths. It can be seen from Figure 9 that all the axial stress-axial strain curves of FRP-confined concrete in the test columns feature approximately a bilinear shape with two segments. The stress-strain curves show monotonic ascending, while the stress-strain curves of specimens in Figure 9a (specimens wrapped with three strips) show descending second-segment. The descending stress-strain behavior is resulted from the increase in clear spacing of adjacent FRP strips.

It is seen from Figure 9 that the peak stress increases with the width of FRP strips while the slope of the second-segment seems to be independent of the width of FRP strips. This is because an increase of width of FRP strips leads to a decrease of clear spacing of adjacent FRP strips, which subsequently leads to an increase in the confinement stiffness. It is also found from Figure 9 that the ultimate axial strain of concrete and hoop rupture strain of FRP were independent of the clear spacing of adjacent FRP strips, which is in accordance with the findings by Zeng et al. [15]. This implies difference between partially FRP confined-concrete and fully FRP confined-concrete, as the ultimate axial strain was found to be highly dependent on the FRP volume ratio. The details of the ultimate conditions will be discussed in detail in the following section. It is interesting to find that the peak axial stress increases with the FRP strip thickness while the slope of the second-segment is nearly independent of the strip thickness.

The peak axial stresses ($f_{\mathrm{cc}}^{\prime }),$ axial strains at peak stresses ($\varepsilon_{\mathrm{cc}}$), ultimate axial stresses ($f_{\mathrm{cu}}^{\prime }$), ultimate axial strains ($\varepsilon_{\mathrm{cu}})$ and FRP hoop rupture strains ($\varepsilon_{h,\mathrm{rup}}$) (at the mid-height section) for the test columns are listed in Table 1. The normalized ultimate axial stresses ($f_{\mathrm{cu}}^{\prime }/f_{\mathrm{co}}^{\prime }$), normalized ultimate axial strains ($\varepsilon_{\mathrm{cu}}/\varepsilon_{\mathrm{co}}$) are also given in Table 1. In Table 1, $\varepsilon_{h,\mathrm{rup}}$ refers to the average FRP hoop strain from the two hoop strain gauges which were outside the overlap. It is noteworthy that the ultimate axial stress is equal to the peak stress for the confined-concrete with sufficient FRP confinement (i.e., the stress-strain behavior is characterized by a monotonic ascending branch); in this case only ultimate axial stress and ultimate axial strain are given in Table 1. However, as for the confined concrete with insufficient FRP confinement (i.e., the stress-strain behavior is generally characterized by an ascending first branch and a second descending branch), the ultimate axial stress is smaller than the peak strength; in this case both the ultimate and peak axial stresses and corresponding strains are given. The average FRP hoop rupture strain $\varepsilon_{h,\mathrm{rup}}$ was found to be independent of the FRP strip layers and width, as given in Table 1. As shown in Table 1, the normalized ultimate axial stresses ($f_{\mathrm{cu}}^{\prime }/f_{\mathrm{co}}^{\prime }$) and normalized ultimate axial strains ($\varepsilon_{\mathrm{cu}}/\varepsilon_{\mathrm{co}}$) varied in a considerable range with respect to the column parameters. It is found from Table 1 that the normalized axial stresses range from 0.93–1.81 and normalized ultimate axial strains are from 2.88–13.12, which demonstrates excellent strength and ductility of partially FRP-confined concrete. The normalized ultimate axial stresses of specimens that experienced descending stress-strain curves were less than one. This is believed to be due to the existence of a larger clear strip spacing of these specimens, which easily leads to localized crushing failure of concrete.

### 3.3. Axial and Hoop Strain Distribution

**Figure 10:**
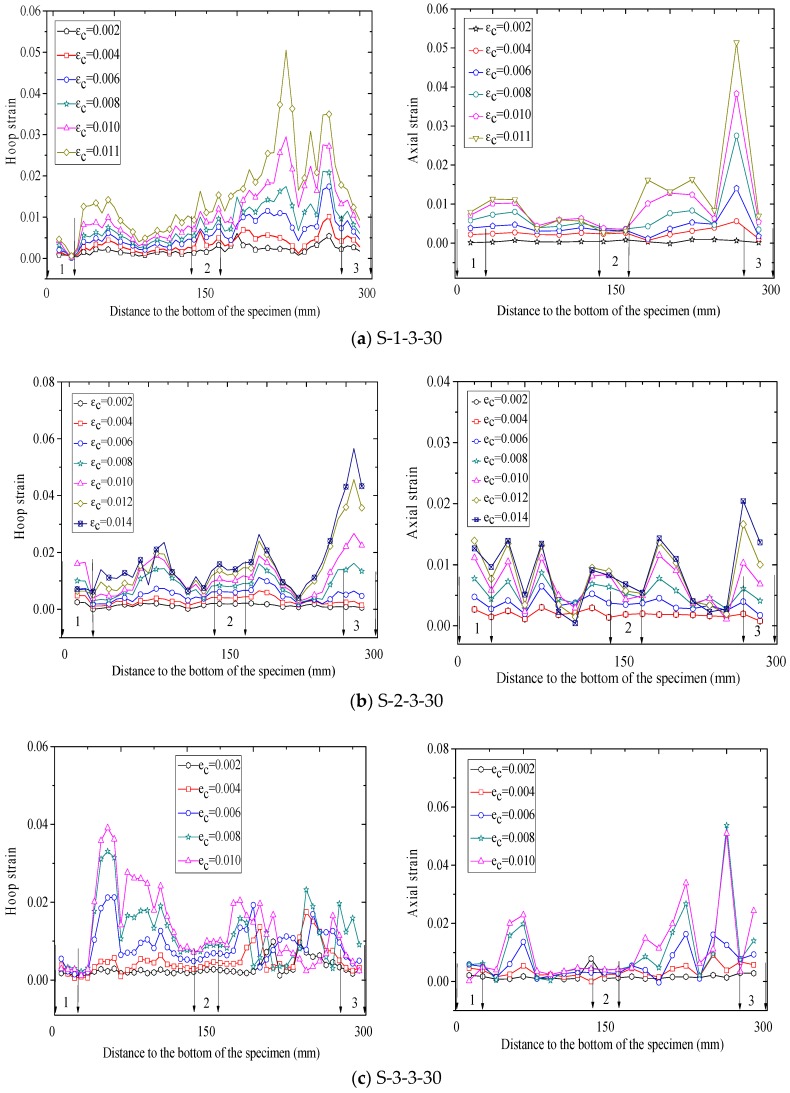
Strain distribution along the column height (specimens wrapped with three strips).

**Figure 11:**
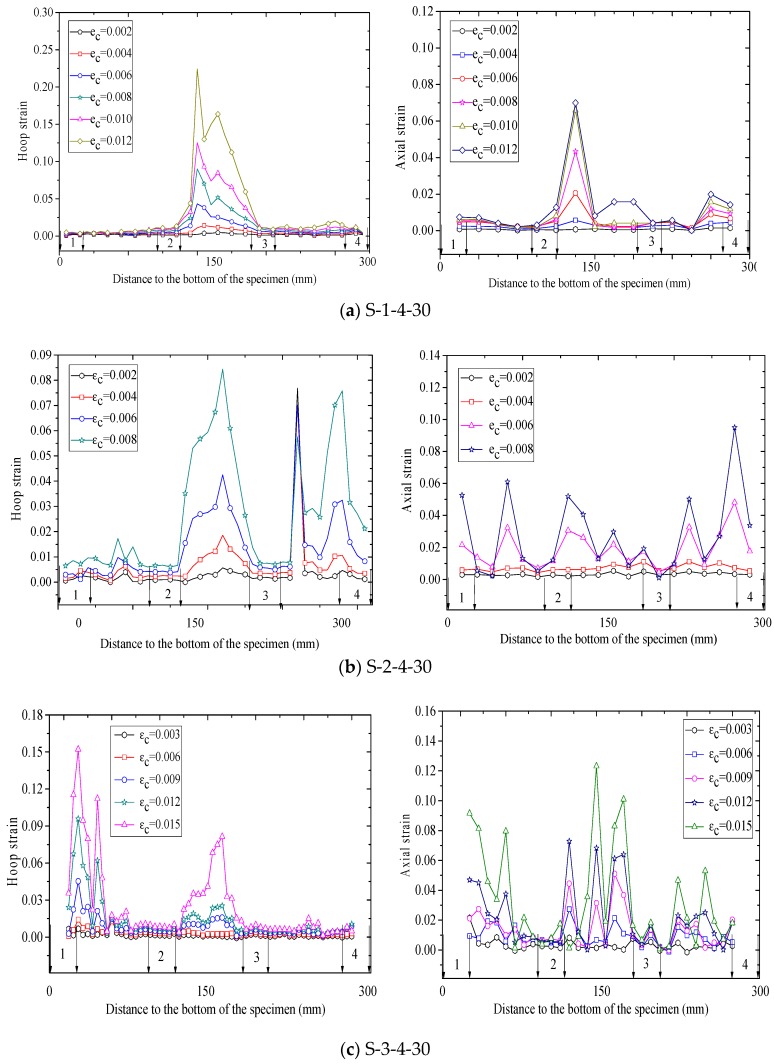
Strain distribution along the column height (specimens wrapped with four strips).

**Figure 12:**
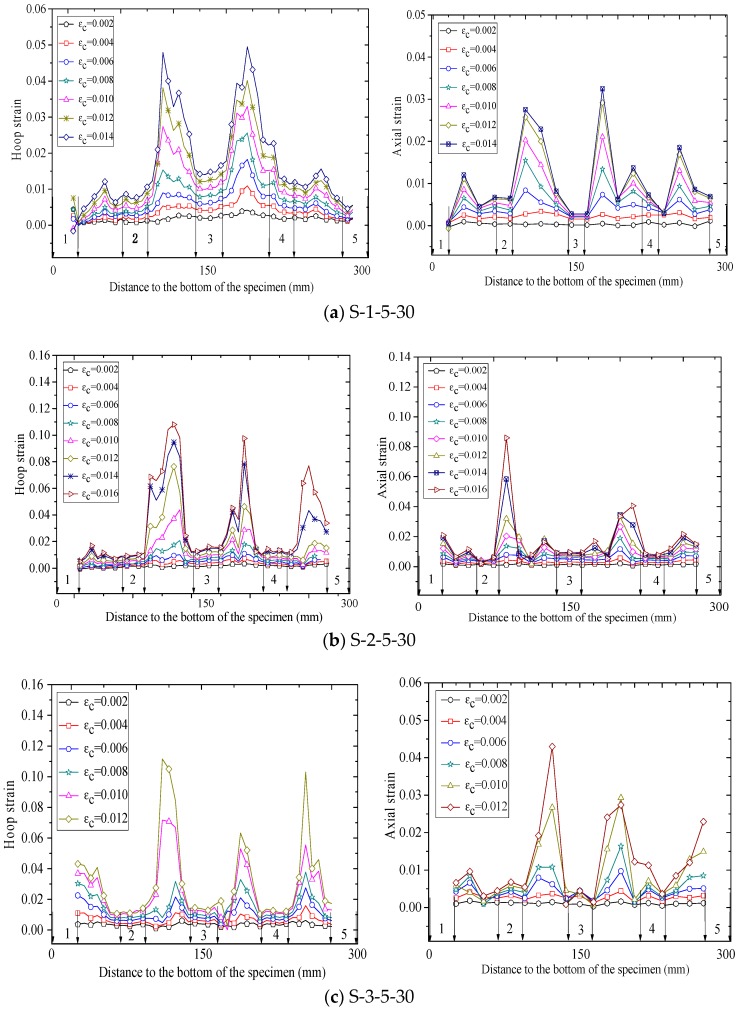
Strain distribution along the column height (specimens wrapped with five strips).

Figure 10, Figure 11 and Figure 12 show revolutionary insights into the distribution of hoop strains and axial strains along the height of the specimens at different axial strain levels, with the strains being drawn in the vertical axis and the distance to the bottom of the specimen being drawn in the horizontal axis. Note that in Figure 10, Figure 11 and Figure 12, the number between two arrows indicates the location of FRP strip. The axial and hoop strain distributions were based on the PIV technique and the virtual strain gauges were located opposite to the center of the overlap layer (Figure 6). The gauge lengths of the virtual strain gauges were 20 mm for both hoop strain and axial strain measurement.

The evolution of hoop strains appears to increase consistently at all displacement levels, providing additional credence to the optical strain measurement technique. It is interesting to find that the axial strains and hoop strains are larger at the mid-plain of the two adjacent FRP strips than those at the mid-plain of each FRP strip. This is because the confining stress to concrete between the FRP strips is less effective than that of concrete in the FRP strips at a given axial strain, which leads to a larger axial strain in the concrete between the FRP strips. This serves as a reason why ultimate axial strain of partially FRP-confined concrete based on LVDTs is larger than that of fully FRP-confined concrete [15]. In most circumstance, hoop strains in FRP jacket remained small and uniform along the height until the average axial stress of concrete surpassed the unconfined concrete strength, after which the hoop strains increased rapidly as the confined concrete began to dilate, activating the FRP confinement. Figure 10, Figure 11 and Figure 12 also clearly show that the hoop strain distribution is uniform along the height at strain levels below 0.004, while the hoop strains in FRP strips are substantially smaller than those in the concrete between adjacent strips when the axial strain was larger than 0.004. This is because the cracking of the concrete at/near the mid-plain of the two adjacent FRP strips leads to substantial increase of hoop strains.

Considerable variation is observed in virtual axial strain readings over the height of specimens, although the upper half of the specimen are symmetrical to the bottom half. Additionally, the hoop strain distributions are not vertically symmetrical, although the column is symmetrical in the vertical direction. These hoop strain distributions also provide a striking explanation for the observed variation in hoop strain measurement which is implicit in essentially all previously published research on FRP-confined concrete. It also implies that it is not surprising that the maximum recorded hoop stains by conventional foil strain gauges at failure show strain efficiency values with substantial inconsistency. Nevertheless, the existing available test data of hoop strain on an FRP-confined concrete member is mainly depended on conventional foil strain gauges. It is, therefore, not at all surprising that the available model for predicting FRP-confined concrete’s ultimate axial strain are incapable of making predictions with an average absolute error of less than about 20% [61].

## 4. Finite-Element Modeling of FRP-Confined Concrete

### 4.1. General

An improved versatile constitutive concrete damage-plastic model (CDPM) which can produce accurate predictions for FRP-confined concrete under both uniform and non-uniform confinement was proposed and carefully validated by Yu et al. [41]. In this model, all the material parameters have been found from Teng et al.’s analysis model [62], which is for concrete under uniform FRP confinement. In Yu et al. [41]’s model, an effective confining pressure was defined and two methods of defining the flow rule were proposed for non-uniformly confined concrete. It was found that this model is capable of providing reasonable stress-strain responses for FRP-confined concrete in both uniform and non-uniform sections by subsequent investigations [40,41,44,63]. For ease of reference, Yu et al. [41] model is briefly summarized in the following sections.

### 4.2. FRP-Confined Concrete

As explained earlier, the modified CDPM [41] has related the loading function *F*, the hardening function κ, the potential function G, and the damage parameter *d* to confinement-dependent variables. The loading function *F* adopts the yield function proposed by Lubliner et al. [64] and modified by Lee and Fenves [65]. As can be found in the ABAQUS theory manual, the mathematic expression for *F* is:

(1) $$F=\frac{1}{1-A}\left(\sqrt{3{\bar{J}}_{2}}-A{\bar{I}}_{1}+B\left({\tilde{\varepsilon }}_{p}\right)\langle -{\bar{\sigma }}_{\min}\rangle -C\langle -{\bar{\sigma }}_{\min}\rangle \right)-{\bar{\sigma }}_{\mathrm{cn}}\left({\tilde{\varepsilon }}_{\mathrm{pc}}\right)=0$$

with

(2) $$A=\frac{\frac{f_{b}^{\prime }}{f_{\mathrm{co}}^{\prime }}-1}{2\frac{f_{b}^{\prime }}{f_{\mathrm{co}}^{\prime }}-1};\;0\leq A\leq 0.5,$$

(3) $$B=\frac{{\bar{\sigma }}_{\mathrm{cn}}\left({\tilde{\varepsilon }}_{\mathrm{pc}}\right)}{{\bar{\sigma }}_{\mathrm{tn}}\left({\tilde{\varepsilon }}_{\mathrm{pt}}\right)}\left(1-A\right)-\left(1+A\right),$$

(4) $$C=\frac{3\left(1-K\right)}{2K-1}.$$

where, ${\bar{I}}_{1}$ is the first invariant of the effective stress, ${\bar{J}}_{2}$ is the second invariant of the effective stress, $f_{b}^{\prime }$ is the biaxial concrete strength, $f_{\mathrm{co}}^{\prime }$ is unconfined concrete cylinder strength, ${\bar{\sigma }}_{\min}$ is the minimum principal effective stress, ${\tilde{\varepsilon }}_{p}$ is the equivalent plastic strain, ${\tilde{\varepsilon }}_{\mathrm{pc}}$ is the equivalent compressive plastic strain, ${\tilde{\varepsilon }}_{\mathrm{pt}}$ is the equivalent tensile plastic strain, ${\bar{\sigma }}_{\mathrm{cn}}$ is the effective compressive cohesive strength, ${\bar{\sigma }}_{\mathrm{tn}}$ is the effective tensile cohesive strength, K is the ratio of biaxial concrete strength to triaxial compression strength and the Macauley bracket 〈·〉 is defined by 〈x〉=(|x|+x)/2.

The value 0.725 of K has been found by Yu et al. [44] using the empirical equation for the strength of concrete under biaxial compression and tri-axial compression [62]. Then other parameters need be input for this loading function are the unconfined concrete strength $f_{\mathrm{co}}^{\prime }$ and the ratio of $f_{b}^{\prime }$ to $f_{\mathrm{co}}^{\prime }$ which has a default value 1.16 in the ABAQUS software. The strain hardening rule is defined in the function of ${\bar{\sigma }}_{\mathrm{cn}}\left({\tilde{\varepsilon }}_{\mathrm{pc}}\right)$.

The flow potential function *G* adopted in the CDMP model is the hyperbolic function as follows:

(5) $$G=\sqrt{\left(∋\sigma_{\mathrm{to}}\tan\psi \right)+{\bar{J}}_{2}}-{\bar{I}}_{1}\tan\psi$$

where Ψ is the dilation angle; $\sigma_{\mathrm{to}}$ is the uniaxial tensile stress at failure; ∋ is the eccentricity; when the eccentricity ∋ is zero, Equation (5) degenerate to:

(6) $$G=\sqrt{{\bar{J}}_{2}}-{\bar{I}}_{1}\tan\psi$$

For concrete under uniform confining pressure, the value of tanψ can be obtained using the following equation:

(7) $$\tan\psi =\frac{\sqrt{3}}{6}\frac{d\varepsilon_{c}^{p}+2d\varepsilon_{l}^{p}}{d\varepsilon_{c}^{p}-d\varepsilon_{l}^{p}}$$

where $d\varepsilon_{l}^{p}$ is the plastic strain increment in the lateral direction.

Yu et al. [41] used the following assumption to simplify the input of damage variable. The damage variable is set to be zero before the peak stress and it is selected so that the effective axial stress-strain curve yields a platen after peak stress. That means for concrete under uni-axial compression, the expression of the damage variable is:

(8) $$d=1-\frac{\sigma_{c}}{f_{\mathrm{co}}^{\prime }}$$

Meanwhile for concrete under constant confining pressure, the expression of the damage variable can be revised to:

(9) $$d=1-\frac{\sigma_{c}-\frac{1+C+2A}{1-A}\sigma_{l}}{f_{\mathrm{cc}}^{\prime *}-\frac{1+C+2A}{1-A}\sigma_{l}}$$

where $f_{\mathrm{cc}}^{\prime *}$ is the peak stress of concrete under a constant confining pressure.

Beside the above four components in Yu et al. [41], the following equation was employed to calculate the equivalent confining pressure:

(10) $$\sigma_{l,\mathrm{eff}}=\frac{2\left(\sigma_{2}+0.039f_{\mathrm{co}}^{\prime }\right)\left(\sigma_{3}+0.039f_{\mathrm{co}}^{\prime }\right)}{\sigma_{2}+\sigma_{3}+0.078f_{\mathrm{co}}^{\prime }}-0.039f_{\mathrm{co}}^{\prime }$$

where $\sigma_{l,\mathrm{eff}}$ is the equivalent confining pressure; $\sigma_{2}$, $\sigma_{3}$ are confining pressure in vertical direction.

These modifications were ultimately implemented in the ABAQUS software using the solution dependent field variable (SDFV) option. The inclusion of confinement-dependent features in FE models requires the use of an analysis-oriented stress-strain model for FRP-confined concrete to produce the necessary material parameters, as all the material parameters have been based on analysis-oriental stress-strain model for uniformly confined concrete and it was expected that the FE model has same accuracy as the adopted analysis-oriental stress-strain model [44]. The procedure of generating concrete input data provided by Yu et al. [41] was adopted to generate the concrete field-dependent input data in the ABAQUS.

### 4.3. FRP Jacket

The FRP strip is modeled as a linear elastic brittle material. The stiffness of FRP in the axial direction (i.e., the loading direction) is negligible as the fibers in the FRP jackets are all oriented in the hoop direction. The definition of the elastic behavior of FRP jacket was based on the elastic lamina option in the ABAQUS which is used to model an isotropic elastic material. As explained earlier, the negligibility of FRP axial stiffness was achieved by activated the “no compression” option of elastic material in the ABAQUS. The Poisson’s ratio of FRP was set to be zero.

### 4.4. Modeling of Partially FRP-Confined Circular Columns

**Figure 13:**
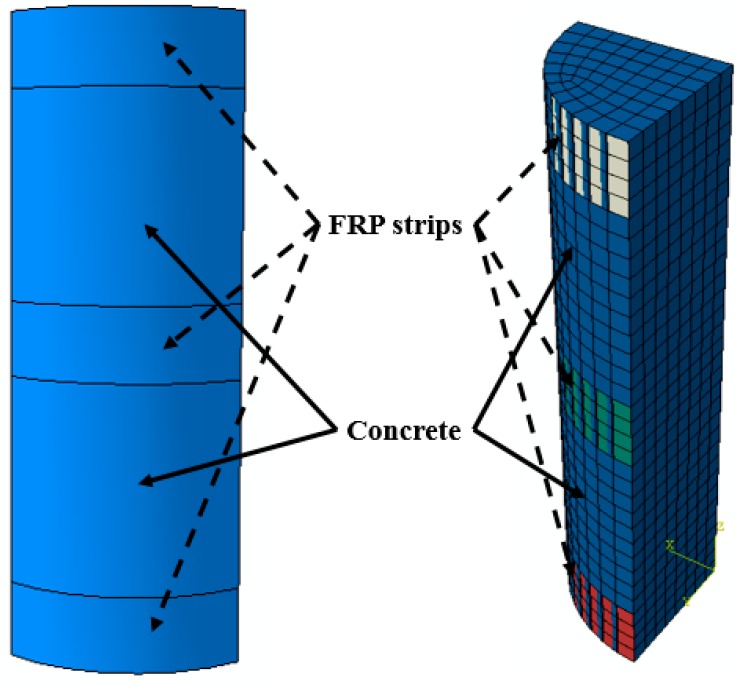
Symmetry FE model for a partially FRP-confined circular column.

**Figure 14:**
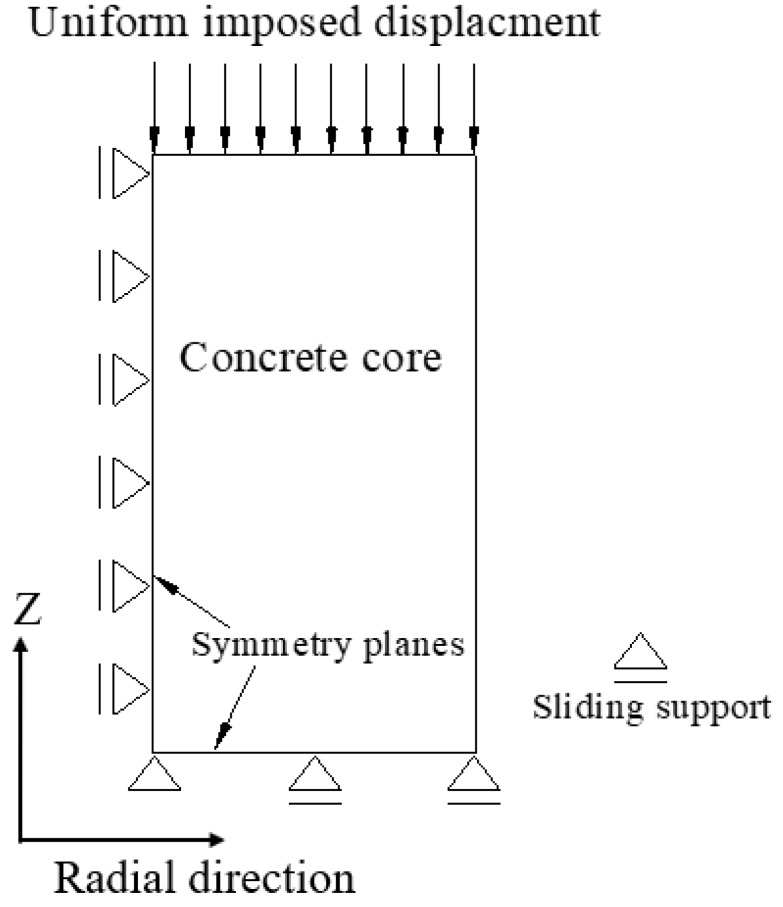
Boundary conditions and loading of the simulated column specimens.

**Figure 15:**
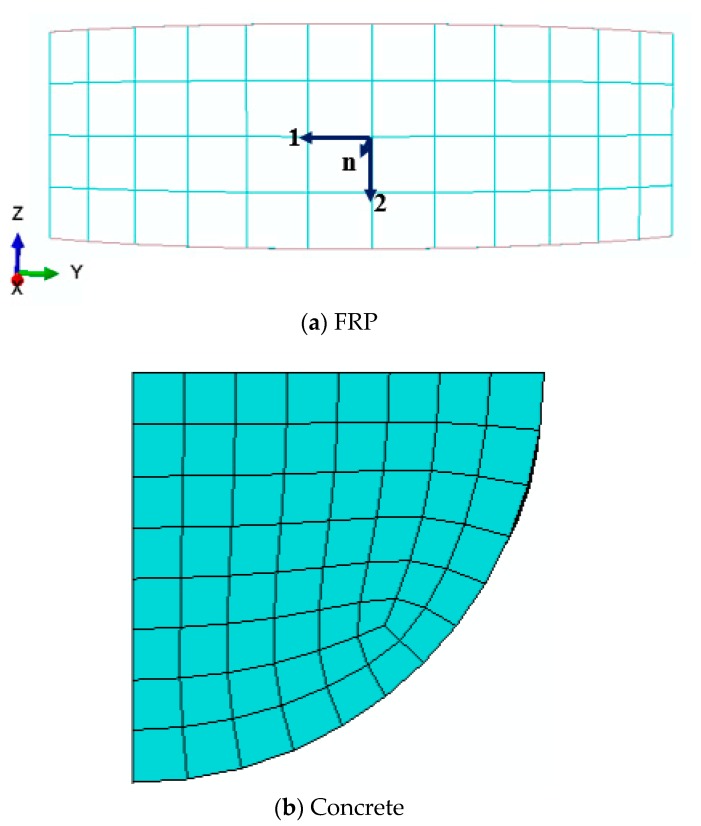
Meshing of the FE model.

For each of column being investigated by FE approach, a one-fourth column model was established due to the symmetrical features of the column. Figure 13 shows the FE model of partially FRP-confined circular column, taking specimen S-1-3-30 as an example. The boundary condition of the FE model is shown in Figure 14, based on the symmetrical boundary of the model of a quarter of column. In the FE model, concrete was modeled using 8-node solid elements (i.e., C3D8R) and FRP was modeled with 4-node shell elements (i.e., S4R). The FRP was tied to concrete using of the ABAQUS tie constraint and the FRP jacket was tied to the corresponding node on the outer surface of the concrete infill so that the two nodes were forced to experience the same translations. A local coordinate system was assigned to the FRP jacket and the hoop direction and the axial direction were adopted as the 1-principan and 2-principal material orientations, respectively (Figure 15a).

The modified concrete damage plasticity model [41], illustrated above, was adopted for the concrete. The unconfined concrete strength $f_{\mathrm{co}}^{\prime }$ and corresponding unconfined concrete strain $\varepsilon_{\mathrm{co}}$ were obtained from compression tests presented in the current study. For each specimen, only a quarter of it was created in the FE model due to its symmetric nature. Mesh convergence studies were conducted for the model until suitable mesh sizes (10 mm) were found (Figure 15b). This provided almost the same axial stress-strain curves as those from the next level of refinement.

### 4.5. Numerical Results

#### 4.5.1. Stress-Strain Curves

**Figure 16:**
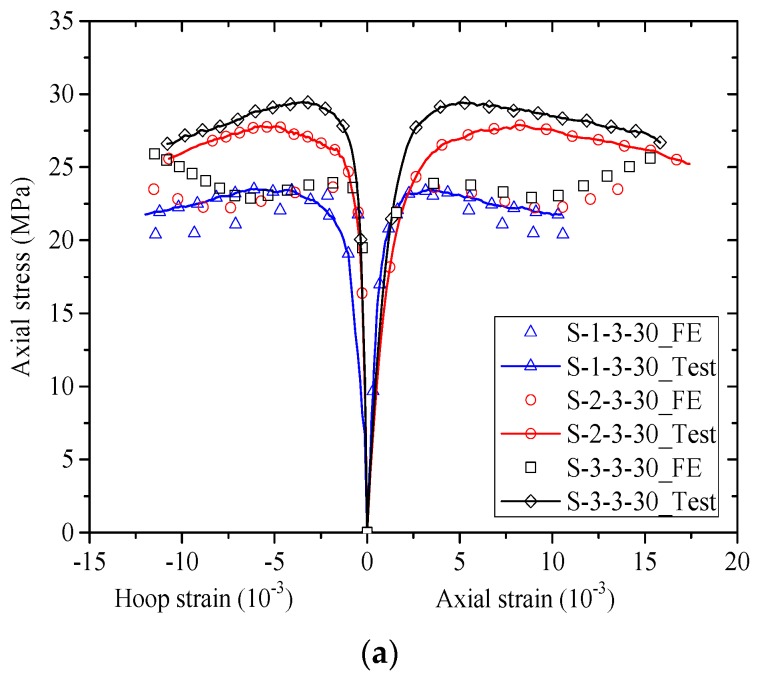

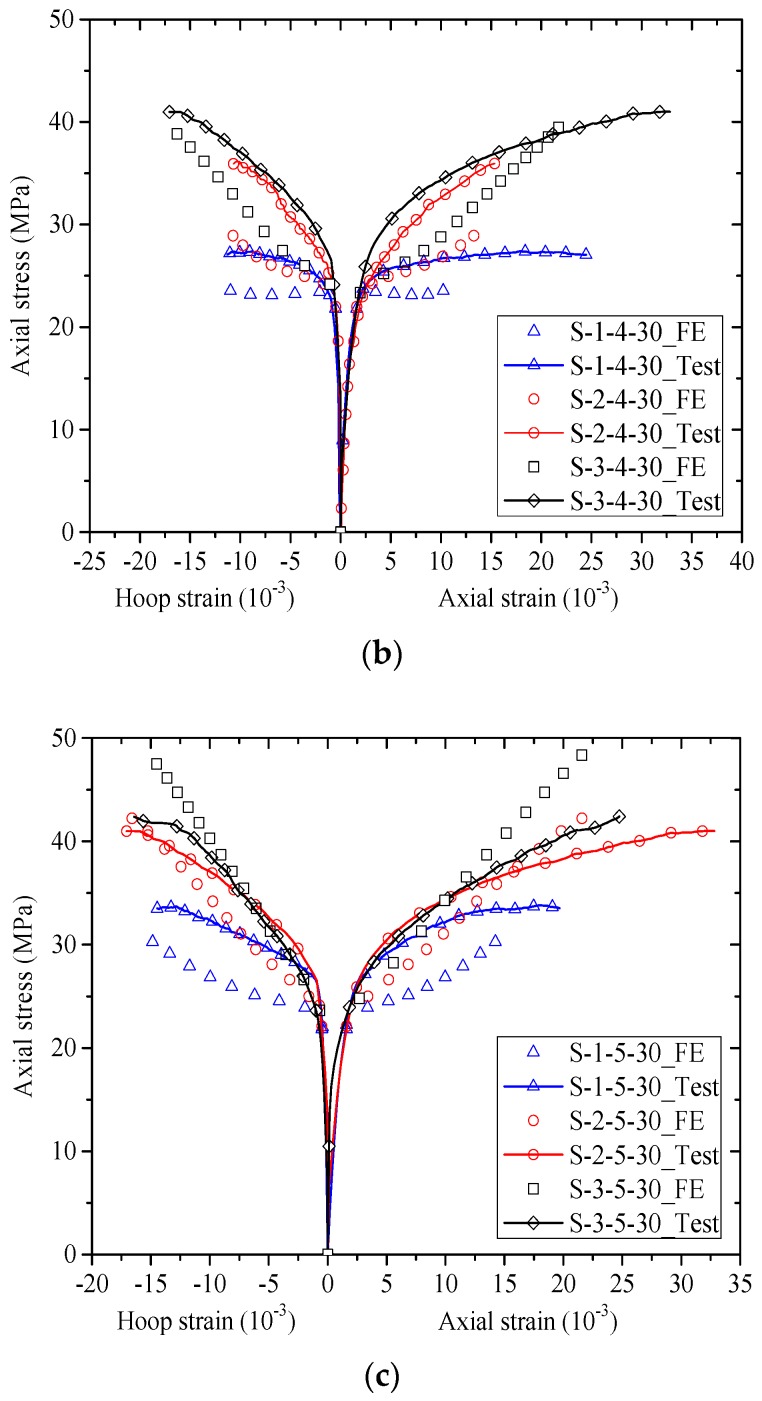
Stress-strain curves (experimental results versus finite element predictions). (**a**) Specimens wrapped with three strips; (**b**) Specimens wrapped with four strips; (**c**) Specimens wrapped with five strips.

Figure 16 shows the comparisons between experimental stress-strain curves and predicted stress-strain curves by the FE approach. Note that the predicted curves were terminated at a hoop strain equal to the average FRP hoop rupture strain based on test results. The test results presented in current paper were utilized to validate the accuracy of the FE approach. It can be seen from Figure 16 that the predicted axial stress-strain curves are lower than the test results. More specifically, it is shown that the FE approach is capable of closely predicting the ultimate axial strain of the test columns while it provides conservative axial compressive strength. The error of the FE approach in predicting ultimate axial stress is within 20% for most of the specimens, which is in accordance with the findings given by Yu et al. [41]. The authors believe that the errors may result from the utilization of effective confining stress for concrete under nonuniform confinement, which was derived by Yu et al. [44] based on data from experimental programs conducted four decades ago. Nevertheless, the FE approach provides conservative results for the compressive strength of some specimens (e.g., S-2-4-30, S-3-5-30). However, for the experimental stress-strain curves of these specimens (i.e., S-2-4-30, S-3-5-30), the slope of the second-segment seems to be independent of the FRP strip thickness, which contradicts to the commonly accepted experimental observation reported by other researchers that a higher FRP confinement stiffness leads to a higher slope of the second-segment. This implies it is reasonable to believe that the errors in predicting the above two specimens have little implication for the comparison.

#### 4.5.2. Distribution of Axial Stresses and Radial Stresses

**Figure 17:**
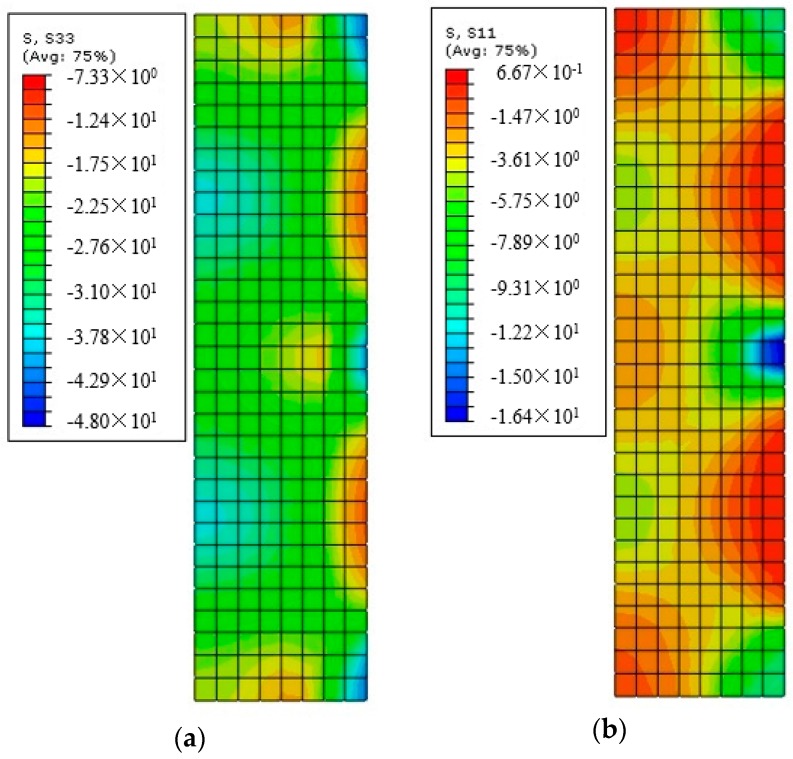
Stress distribution in shaft section (*X*-*Z* section) of S-2-3-30 ($\varepsilon_{h,\mathrm{frp}}$ = 1.5%). (**a**) Axial stress; (**b**) Radial stress.

Figure 17 shows the FE axial stress and radial stress distributions in the shaft (axial-radial) section of S-2-3-30 ($\varepsilon_{h}$ = 1.5%). This is served as an example of the axial stress and radial stress distributions in concrete in circular columns confined with FRP strips and the axial stress and radial stress distributions of specimens with different FRP strip thicknesses and widths are not given in the current paper to limit the length of it. It is seen from Figure 17 that both the axial stress and radial stress at the mid-plain of each FRP strip level are larger than those at the mid-plain level of the two adjacent FRP strips. This suggests the plausibility of “arching action” between the levels of transverse hoop reinforcement [28,29]. It was believed that at the mid-plain of the two adjacent FRP strips (Figure 3), the area of ineffectively confined concrete will be largest and the area of effectively confined concrete core will be smallest. The axial stress distribution shown in Figure 17 clearly demonstrates the arching action in concrete confined with FRP strips. It should be noted that the arching action is suggested for the FRP effective confinement pressure in the partially FRP-confined columns in the existing codes (e.g., [26,27]), in which it is assumed that the hoop confining pressure is only effective for concrete in the effective confinement area where the confining pressure has fully developed due to the arching action.

**Figure 18:**
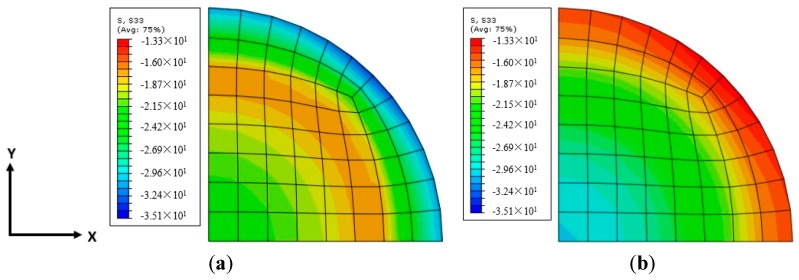
Axial stress distribution in the horizontal section (*X*-*Y* section) of specimen S-2-3-30 ($\varepsilon_{h,\mathrm{frp}}$ = 1.5%). (**a**) Mid-plain of each FRP strip; (**b**) Mid-plain of two adjacent FRP strips.

**Figure 19:**
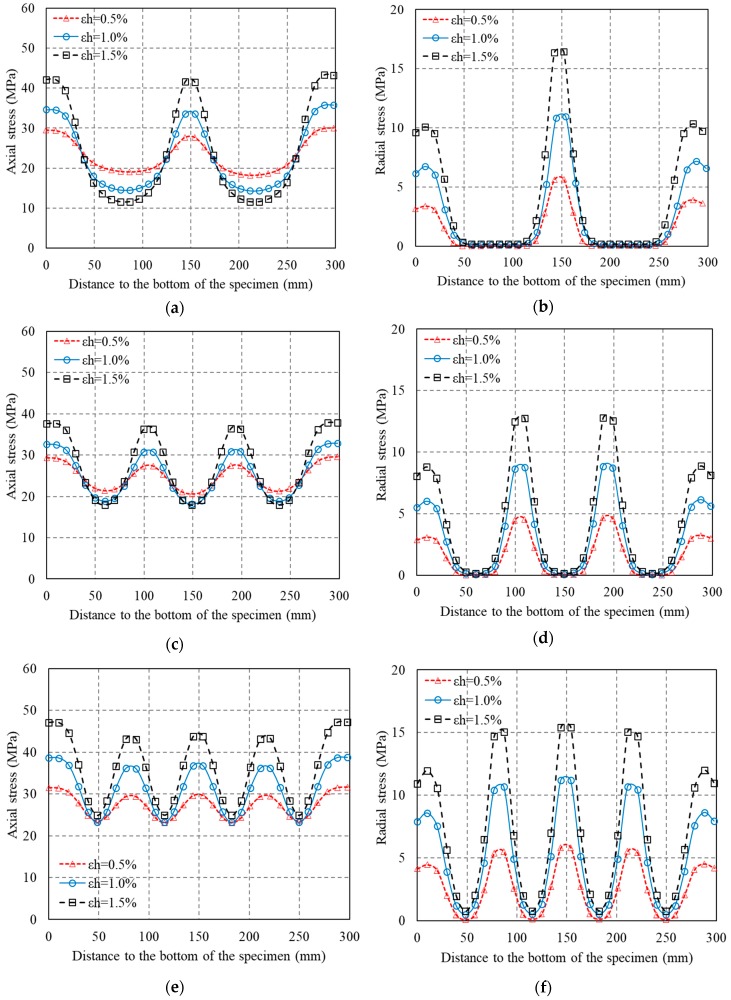
Axial stress and radial stress distributions along the generatrix (column height). (**a**) Axial stress (S-2-3-30); (**b**) Radial stress (S-2-3-30); (**c**) Axial stress (S-2-4-30); (**d**) Radial stress (S-2-4-30); (**e**) Axial stress (S-2-5-30); (**f**) Radial stress (S-2-5-30).

The axial stress distribution over the section (a quarter of the horizontal section) is shown in Figure 18. In the mid-plain of each FRP strip, the axial stress distribution along the radial direction is more uniform than that in the mid-plain of the two adjacent FRP strips, indicating that the area of ineffectively confined concrete is largest and the area of effectively confined concrete core is smallest at the mid-plain of the two adjacent FRP strips. Figure 19 shows the distributions of axial stress and radial stress at the section edge along the generatrix (column height) at three given FRP hoop strain levels (i.e., 0.5%, 1.0% and 1.5%) for specimens wrapped with two layers of 30-mm FRP strips. It is obvious that at the mid-plain of each FRP strip, both the axial and radial stresses at the section edge are largest than those at the mid-plain of the two adjacent FRP strips. This is in accordance with the experimental observations. The deviation of radial stress of concrete increases with an increase in FRP hoop strain. This indicates that the confining stress becomes much more nonuniform with the increase of concrete expansion.

**Figure 20:**
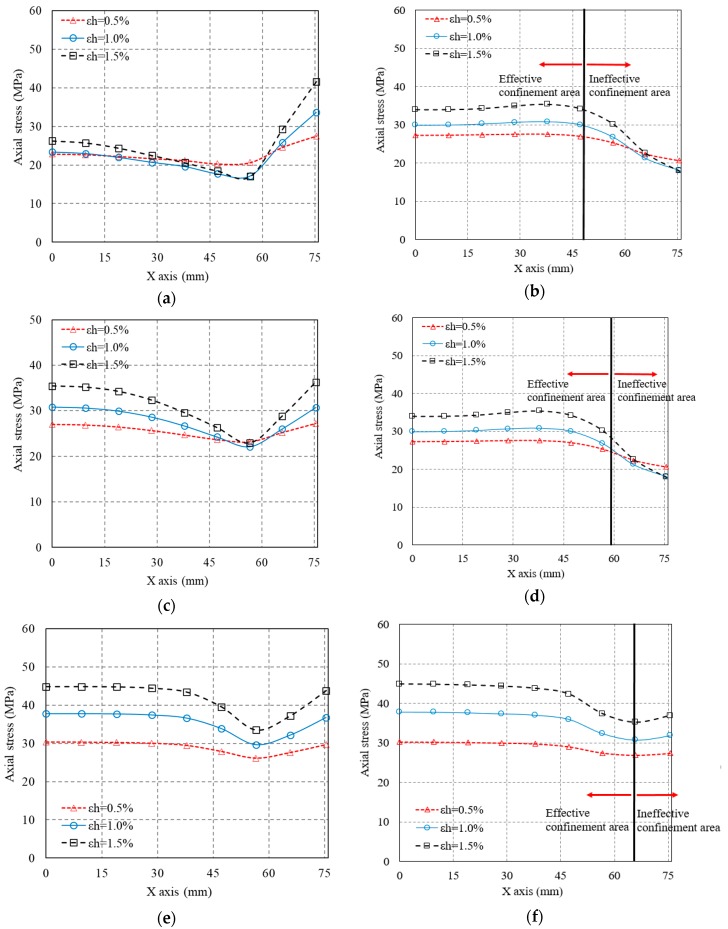
Axial stress distribution along the radial direction (*X* axis). (**a**) Mid-plain of each FRP strip (S-2-3-30); (**b**) Mid-plain of two adjacent FRP strips (S-2-3-30); (**c**) Mid-plain of each FRP strip (S-2-4-30); (**d**) Mid-plain of two adjacent FRP strips (S-2-4-30); (**e**) Mid-plain of each FRP strip (S-2-5-30); (**f**) Mid-plain of two adjacent FRP strips (S-2-5-30).

To further validate the arching action, the axial stress distribution along the radial direction (*X* axis) is given in Figure 20, with the axial stresses being drawn in the vertical axis and the radial coordinate being drawn in the horizontal axis. It is also obvious that in the mid-plain of each FRP strip, the axial stress distribution along the radial direction is more uniform than that in the mid-plain of the two adjacent FRP strips. In Figure 20, a solid line was added to each figure to indicate the locations of ineffective and effective confinement areas. It is found that the axial stress experiences a quick drop after the radial coordinate value approximately reach $D-s_{f}^{\prime }/2$ (Figure 20). This provides an evidence for the determination of diameter of effective confinement core, which is believed to be equal to the section dimension minus half of the clear spacing of the two adjacent FRP strips (Figure 2) [28].

#### 4.5.3. Distribution of Axial Strains and Hoop Strains

**Figure 21:**
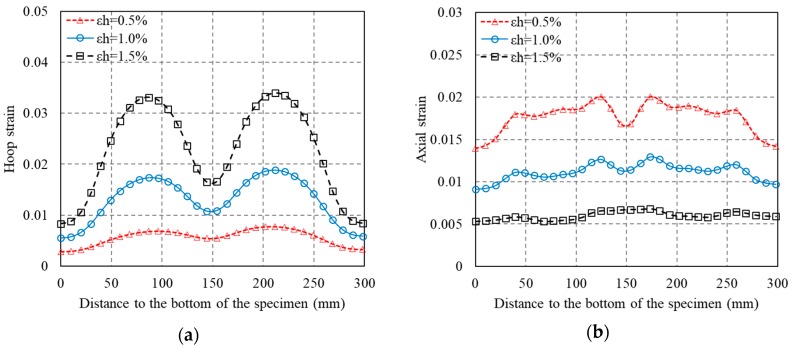

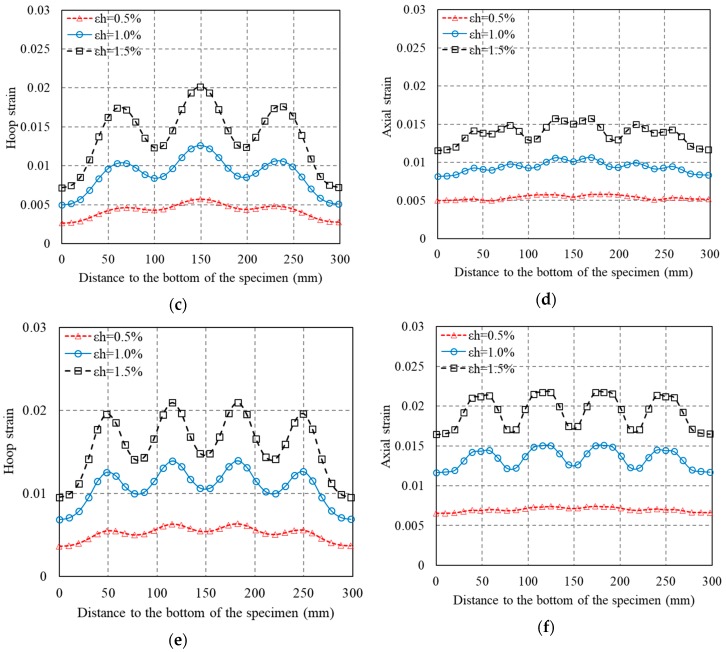
Axial strain and radial strain distribution along the generatrix (column height). (**a**) Hoop strain (S-2-3-30); (**b**) Axial strain (S-2-3-30); (**c**) Hoop strain (S-2-4-30); (**d**) Axial strain (S-2-4-30); (**e**) Hoop strain (S-2-5-30); (**f**) Axial strain (S-2-5-30).

Figure 21 shows the axial and hoop strain distributions of concrete confined with FRP strips along the generatrix (column height) of the specimens. The following three findings can be observed from Figure 21: (1) the trend of axial strain and hoop strain distributions provided by the FE approach are in accordance with the experimental observations: the axial strain and hoop strain at the mid-plain of the two adjacent FRP strips are larger than those at the mid-plain of each FRP strip; (2) the predicted axial strains and hoop strains in the FRP strips are in reasonable agreement with test results while it is seen that the strains between FRP strips deviate substantially from the test results. Above findings from the numerical results indicate a confinement mechanics difference between partially FRP-confined concrete and fully FRP-confined concrete.

For the first finding, it is not surprising that both the axial strain and hoop strain at the mid-plain of the two adjacent FRP strips are larger than those at the mid-plain of each FRP strip as the concrete between FRP strips is easier to experience crushing failure. This clearly demonstrates an experimental observation that the ultimate axial strain in partially FRP-confined concrete is larger than that in fully FRP-confined concrete if the axial strain is averaged from mid-height axial shortening. This also implies the ultimate axial strain of partially FRP-confined concrete will be underestimated by the analysis-oriented model [62] which is based on fully FRP-confined concrete while this phenomenon needs further experimental and theoretical verifications. For the second finding, the measured strains in concrete become unreliable when the concrete spalling occurred. This explains why the predicted axial strains and hoop strains between FRP strips deviate substantially from the test results.

Parametric studies on partially FRP-confined concrete based on the FE approach can be conducted to gain in-depth understandings on the confinement mechanics. Further research on confinement mechanics of partially FRP-confined concrete wrapped with continuous FRP strip spirals is necessary. To validate the FE approach, pressure measurement technique (e.g., [63]) is favored to measure the stress distribution in partially FRP-confined concrete.

## 5. Conclusions

Test results based on the 15 FRP-strengthened cylindrical concrete column partially wrapped with FRP strips have been presented in this paper. Novel PIV technique was adopted to measure axial and hoop strains in the specimens. Never-seen axial and hoop strain variations along the columns were presented. A finite element model based on concrete damage plasticity model was established and validated by the test results. The confinement mechanics of partially FRP-confined concrete was then investigated by the FE approach. Based on the combined test results and numerical results, the following conclusions were drawn:

(1) The peak stress of confined concrete increases with an increase in width of FRP strip. The ultimate axial strain of concrete, the FRP hoop rupture strain and the slope of the second-segment are independent of the clear spacing of adjacent FRP strips. The compressive strength increases with an increase in the FRP strip thickness while the slope of the second-segment is independent of the strip layer.
(2) Based on digital image correlation technique, it is found that both the axial strains and hoop strains are larger at the mid-plain of the two adjacent FRP strips than those at the mid-plain of each FRP strip.
(3) A finite element model based on modified concrete damage plasticity model was utilized to simulate the partially FRP-confined concrete. it is found that the FE approach is capable of closely predicting the ultimate axial strain and providing conservative axial compressive strength.
(4) Both the axial stress and radial stress at the section edge in the mid-plain of each FRP strip level are larger than those at the mid-plain level of the two adjacent FRP strips. In the mid-plain of each FRP strip, the axial stress distribution along the radial direction is more uniform than that in the mid-plain of the two adjacent FRP strips, indicating that the area of ineffectively confined concrete is largest and the area of effectively confined concrete core is smallest at the mid-plain of the two adjacent FRP strips. This also suggests that the plausibility of the arching action assumption.
(5) Both the axial and radial stresses in the mid-plain of each FRP strip are larger than those in the mid-plain of each FRP strip. The deviation of radial stress of concrete increases with an increase in FRP hoop strain. This indicates that the confining stress becomes much more nonuniform with the increase of concrete expansion.
(6) The trend of axial strain and hoop strain distributions provided by the FE approach are in accordance with the experimental observations: the axial strain and hoop strain at the mid-plain of the two adjacent FRP strips are larger than those at the mid-plain of each FRP strip.

## Figures and Tables

**Table 1:** Key information of test columns and key test results.

Specimen	$t_{f}$ (mm)	$s_{f}^{\prime }$	$\rho_{f}$	$f_{cc}^{\prime }$ (MPa)	$\varepsilon_{\mathrm{cc}}$	$f_{cu}^{\prime }$ (MPa)	$\varepsilon_{\mathrm{cu}}$	$f_{\mathrm{cu}}^{\prime }/f_{\mathrm{co}}^{\prime }$	$\varepsilon_{\mathrm{cu}}/\varepsilon_{\mathrm{co}}$	$\varepsilon_{h,\mathrm{rup}}$
S-1-3-25	0.167	112.5	0.0008	23.49	0.0030	22.0	0.0072	0.94	2.88	−0.0083
S-1-3-30	0.167	110	0.0010	23.46	0.0033	21.8	0.0110	0.93	4.40	−0.0119
S-1-3-35	0.167	97.5	0.0012	24.30	0.0029	23.0	0.0119	0.98	4.76	−0.0103
S-2-3-30	0.334	110	0.0021	29.44	0.0049	26.7	0.0158	1.14	6.32	−0.0119
S-3-3-30	0.501	110	0.0029	27.87	0.0083	25.4	0.0174	1.09	6.96	−0.0107
S-1-4-25	0.167	66.67	0.0012	/	/	24.5	0.0103	1.05	4.12	−0.0099
S-1-4-30	0.167	60	0.0015	/	/	27.1	0.0245	1.16	9.80	−0.0113
S-1-4-35	0.167	53.33	0.0018	/	/	30.5	0.0169	1.30	6.76	−0.0141
S-2-4-30	0.334	60	0.0030	/	/	33.2	0.0116	1.42	4.64	−0.0072
S-3-4-30	0.501	60	0.0045	/	/	41.0	0.0328	1.75	13.12	−0.0171
S-1-5-25	0.167	43.75	0.0016	/	/	29.8	0.0186	1.27	7.44	−0.0179
S-1-5-30	0.167	37.5	0.0020	/	/	33.5	0.0197	1.43	7.88	−0.0145
S-1-5-35	0.167	31.25	0.0024	/	/	36.7	0.0188	1.57	7.52	−0.0184
S-2-5-30	0.334	37.5	0.0040	/	/	42.4	0.0247	1.81	9.88	−0.0164
S-3-5-30	0.501	37.5	0.0059	/	/	41.0	0.0328	1.75	13.12	−0.0171

Note: $t_{f}$—FRP thickness; $s_{f}^{\prime }$—clear spacing of two adjacent FRP strips; $\rho_{f}$—FRP volumetric ratio.
